# The Molecular Pharmacology of Phloretin: Anti-Inflammatory Mechanisms of Action

**DOI:** 10.3390/biomedicines11010143

**Published:** 2023-01-06

**Authors:** Solomon Habtemariam

**Affiliations:** Pharmacognosy Research & Herbal Analysis Services UK, University of Greenwich, Central Avenue, Chatham-Maritime, Kent ME4 4TB, UK; s.habtemariam@herbalanalysis.co.uk; Tel.: +44-208-331-8302/8424

**Keywords:** phloretin, phlorizin, apple flavonoids, inflammation, Nrf2, NF-κB, cytokines, COX, iNOS

## Abstract

The isolation of phlorizin from the bark of an apple tree in 1835 led to a flurry of research on its inhibitory effect on glucose transporters in the intestine and kidney. Using phlorizin as a prototype drug, antidiabetic agents with more selective inhibitory activity towards glucose transport at the kidney have subsequently been developed. In contrast, its hydrolysis product in the body, phloretin, which is also found in the apple plant, has weak antidiabetic properties. Phloretin, however, displays a range of pharmacological effects including antibacterial, anticancer, and cellular and organ protective properties both in vitro and in vivo. In this communication, the molecular basis of its anti-inflammatory mechanisms that attribute to its pharmacological effects is scrutinised. These include inhibiting the signalling pathways of inflammatory mediators’ expression that support its suppressive effect in immune cells overactivation, obesity-induced inflammation, arthritis, endothelial, myocardial, hepatic, renal and lung injury, and inflammation in the gut, skin, and nervous system, among others.

## 1. Introduction

**Figure 1:**
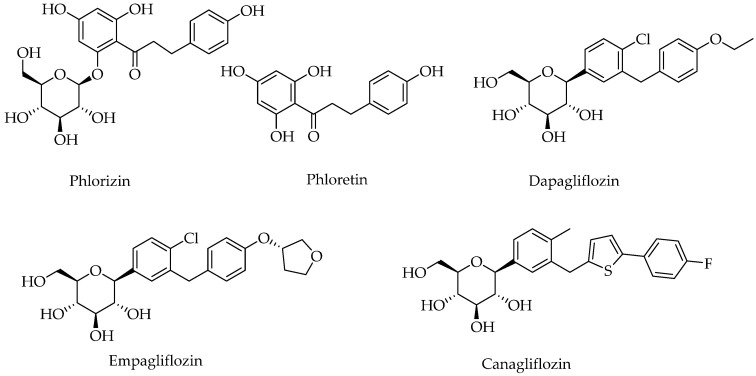
Structures of phlorizin, phloretin, and some selective SGLT2 inhibitors.

Phlorizin (Figure 1) is a naturally occurring dihydrochalcone glycoside first isolated in 1835 as a bitter taste substance from the root bark of an apple (*Malus domestica* Borkh.) tree. As a major phenolic constituent, it is found in the various tissues of the plant (primarily in the young shoots, roots, leaves, bark, and seeds) and its concentration in the leaves can be as high as 14% of the dry weight depending on the cultivar [1]. Known by many common names such as phloridzin, phloretin 2′-*O*-glucoside, phlorrhizin, phlorhizin, or phlorizoside, the compound has been shown to display numerous pharmacological effects of which antidiabetic properties are the most studied. It is a potent inhibitor of the sodium-glucose cotransporter-2 (SGLT2 in the nanomolar range) which is a low-affinity/high-capacity transporter. Though at lower efficacy, it also inhibits SGLT1 (high affinity/low-capacity transporter), and the compound is now known as a dual SGLT1 and SGLT2 inhibitor, with the ranking order of inhibition of SGLTs as SGLT2 > SGLT1 > SGLT4 > SGLT3 >> *v*SGLT [2]. The SGLT2 and SGLT1 transporters are expressed at the apical membranes of polarized epithelial cells and transport glucose against concentration gradients through a mechanism driven by Na^+^ gradients. Most of the dietary glucose uptake from the intestine is mediated through the action of the SGLT1 which is expressed at the brush border membrane of enterocytes, while the SGLT2 accounts for most of the glucose reuptake in the convoluted proximal tubules of the kidney with the rest of the reabsorption taken care of by SGLT1. The required Na^+^ electrochemical gradient is maintained by its active removal on the basolateral end via the action of Na^+^/K^+^-ATPase. In terms of expression, the early proximal tubule (S1/S2 segment), where most of the glucose reabsorption occurs (~97% of fractional glucose reabsorption), expresses SGLT2 while the latter part of the proximal tubule (S2/S3 segments) expresses SGLT1 [3,4]. It has long been established that oral administration of phlorizin induces urinary glucose excretion, renal glycosuria, and weight loss without hyperglycaemia [5,6,7,8,9]. It can also be applied in combination with other antidiabetic agents such as metformin for effective management of glucose metabolism [10]. The presence of the glucose moiety in the structure of phlorizin (Figure 1) is an important structural feature for the observed antidiabetic properties as its aglycone, phloretin, is by order of magnitude less potent than phlorizin. One major drawback in using phlorizin as an orally available antidiabetic agent is however its hydrolysis by intestinal glucosidases (β-glucosidase enzymes) leading to the formation of phloretin: i.e., poor oral bioavailability is a therpeutic limitation of phlorizin. Phlorizin had also been reported to induce renal impairment, an adverse effect in the gastrointestinal system, and other organs where SGLT1 is a transporter such as in the heart [11]; it also inhibits GLUT transporters, (e.g., in the CNS), a function likely to be associated with the hydrolysis product, phloretin. Hence, the contribution of phlorizin in diabetes therapy has mainly been as a pioneer drug for the generation of selective SGLT2 inhibitors, the gliflozins (e.g., dapagliflozin, canagliflozin, and empagliflozin). The selectivity of empagliflozin to SGLT2 over SGLT1 has been reported to be over 2000-fold, dapagliflozin over 1000-fold, and canagliflozin over 200-fold [12].

Given that the sugar moiety of phlorizin is critical to the blockade of the glucose transporters (SGLTs), the stronger effect of phlorizin than its aglycone both as SGLT inhibitor and as an antidiabetic agent [13] is not surprising. The hydrolysis of phlorizin in the small intestine, however, suggests that at least some of the numerous biological activities reported for phlorizin is mediated through its metabolic product, phloretin. Studies also suggest that even the antidiabetic effect of phloretin such as in diabetic nephropathy is mediated through a non-hypoglycaemic effect [14]. Furthermore, phloretin on its own has been shown to display numerous pharmacological effects including anticancer [15,16,17,18,19,20] and antimicrobial [21,22,23,24,25,26] properties and hepatoprotective and cytoprotective effects [27,28,29,30], among others. As a phenolic compound, its antioxidant effect in cellular, acellular, and animal models has also been the subject of numerous studies. In the present communication, the anti-inflammatory effect of phloretin is scrutinised to highlight its reported effect in various diseases (e.g., antiobesity, cardioprotective effects, diabetes, neuroprotection, etc.) may be mediated by targeting the inflammatory axis of the diseases. For the analysis, literature sourced from Science Direct, Pub Med, and Web of Science under the keyword phloretin or phlorizin or phloridzin (years 2000 to 2022) were filtered for inflammation or anti-inflammatory study.

## 2. Macrophages and Other Immune Cells Activation

Macrophages play a critical role both in the initiation and resolution of inflammation. They are involved in antigen presentation, phagocytosis of foreign bodies including pathogens and dead cells, and immunomodulation through the production of proinflammatory and anti-inflammatory cytokines, growth factors, and other small- or large-molecular weight mediators. As a result, most of the studies on the anti-inflammatory effect of phloretin employed macrophage function either in vitro or in vivo. With respect to the known effect of phloretin on glucose transport, the expression of GLUT1 in lymphocytes and macrophages has been shown to be enhanced when they are activated by pro-inflammatory agents such as lipopolysaccharide (LPS) [31]. Reactive oxygen species (ROS) production and oxidative stress have also been shown to enhance the level of GLUT1 expression in macrophages [32]. In microglia too, treatment with LPS and interferon (IFN) increased GLUT1 expression and STF31 (GLUT1-specific inhibitor) dose-dependently reduced glucose uptake and suppressed the upregulation of inflammatory cytokines including tumour necrosis factor (TNF)-α, interleukin (IL)-1β, and IL-6 [33]. Hence, pathologies associated with inflammation such as systemic lupus erythematosus, psoriasis, and autoimmune disorders exhibit upregulated levels of expression of GLUT1 suggesting GLUT1 inhibitors offer improvement in the severity of such diseases [34]. Songyang et al. [35] studied the GLUT1-induced glycolysis in macrophages and whether inhibition by phloretin could offer protection during acute lung injury in an experimental model induced by LPS. In addition to suppression of the LPS-induced augmented mRNA and protein expression of GLUT1, phloretin administration (10 and 50 mg/kg, orally (p.o.)) could protect against lung injury (pathological markers) and suppress the production of inflammatory markers (mRNA levels of IL-1β, TNF-α, and monocyte chemoattractant protein-1 (MCP-1)). This disease-mediated upregulated expression of GLUT1 was also coupled with increased glycolysis levels in lung tissues in LPS-treated mice, which was again inhibited by phloretin. In LPS-treated macrophages culture derived from the mouth peritoneal cavity, phloretin (10, 25, and 50 μM) could decrease the mRNA level of IL-1β, as well as upregulation of mRNA for GLUT1 (but not GLUT3 and GLUT4). Interestingly, the inhibitory effect of phloretin on the expression of IL-1β and TNF-α in LPS-treated macrophages could be reversed by overexpression of GLUT1. Likewise, glycolysis in LPS-stimulated macrophages could be inhibited by phloretin which could be reversed by GLUT1 overexpression. Hence, the known mechanism of GLUT1 inhibition by phloretin should be considered as one of the mechanisms for its anti-inflammatory effects in macrophages and/or other inflammatory cells.

The in vitro LPS-stimulated macrophages assay model is the standard experimental model to demonstrate the anti-inflammatory properties of potential therapeutic agents. Indeed, both phloretin and phlorizin have been tried out in this model for their potential to suppress the release of inflammatory mediators from LPS-activated cells. When tested at the concentration of 3–100 μM, phlorizin did not suppress the inflammatory response in LPS-stimulated RAW 264.7 cells, while phloretin (10 μM) was able to decrease the level of nitric oxide (NO), prostaglandin E_2_ (PGE_2_), IL-6, TNF-α, inducible nitric oxide synthase (iNOS) and cyclooxygenase-2 (COX-2) [36]. The NO release in LPS-stimulated macrophages is a function of the activation of iNOS, an enzyme that cleaves l-arginine into l-citrulline and NO. Activation of induction of COX-2 in macrophages leads to the release of various lipid mediators including PGE_2_, which is a potent inflammatory mediator and pain stimulus. Hence, the compound inhibits the inflammation-associated expression of both cytokine and non-cytokine mediators. This data was also consistent with others where phloretin was shown to suppress the LPS-induced myeloperoxidase (MPO) and iNOS activity in RAW 264.7 cells [37]. In one experiment [38], the IC_50_ value of 5.2 μM was reported as an inhibitory effect of phloretin in NO production by RAW 264.7 cells which was associated with the inhibition of the LPS-induced iNOS. The effect of phloretin in this assay model for NO release ranged from potent (less than 10 μM) to moderate with IC_50_ values between 10–100 μM [36]. The release of proinflammatory cytokines (IL-1α, IL-1β, IL-6, and IFN-γ) and chemokines (C-C motif *chemokine* Ligand 2 (CCL2), CCL5, macrophage inflammatory protein-1α (MIP-1α), and C-X-C motif Chemokine ligand 5 (CXCL5)) from cultured human peripheral blood mononuclear cells stimulated with LPS was also suppressed by phloretin and other natural antioxidants (e.g., silymarin, hesperetin, or resveratrol) when tested at the concentration of 100 µM [39].

One common link to the release of the above-mentioned proinflammatory mediators from activated macrophages is the nuclear transcription factor κ-B (NF-κB) which regulates the expression of several genes (e.g., chemokines, cytokines, and adhesion molecules) involved in inflammation. For details of NF-κB signalling, readers can refer to the numerous review articles in the field (e.g., [40,41]). The NF-κB represents a family of protein transcription factors such as NF-κB1 (also named p50), NF-κB2 (also named p52), RelA (also named p65), RelB, and c-Rel. The expression of target genes happens when these transcription factors translocate from the cytoplasm to the nucleus and bind (through their transcriptional transactivation domain at their C-terminus) to the κB enhancer region of the DNA. Since the NF-κB proteins are sequestered in the cytoplasm by binding to inhibitory proteins, including inhibitors of κB (IκB) family members (most important is IκBα, others include IκBβ, IκBε, and their precursors), releasing the NF-κB from the complex is a key cell signalling step in inflammatory genes activation. Many agents that activate the inflammatory pathway such as LPS stimulate the degradation of IκBα via the multi-subunit IκB kinase (IKK)-induced phosphorylation. This further triggers ubiquitin-dependent IκBα degradation in the proteasome, thereby allowing the nuclear translocation of NF-κB. The IKK itself is a complex of two kinases (IKKα and IKKβ) and other proteins and constitutes the classic example of the canonical NF-κB pathway of activation which is common to various immunostimulant-based macrophage activation: i.e., the IκBα degradation-execute the canonical activation pathway of NF-κB. A variety of inflammatory stimuli activate the canonical pathway in macrophages, and these include proinflammatory cytokines, pattern-associated molecular patterns (PAMPs), or damage-associated molecular patterns (DAMPs) binding to cognate receptors. The LPS is recognised in macrophages by Toll-like receptors (e.g., TLR4) and its stimulatory effect is orchestrated through the canonical pathway. Other pathways or the noncanonical NF-κB pathway of activation are, however, also known. Activation of macrophages to a phenotypically M1 state leads to the induction of inflammatory mediators (e.g., IL-1, IL-6, IL-12, TNF-α, and chemokines) which promotes the process of inflammation while the M2 phenotype leads to the production of anti-inflammatory cytokines (e.g., IL-10 and IL-13) which promote resolution of inflammation (e.g., during the wound healing stage) [42]. Most of the anti-inflammatory mechanisms of phloretin discussed in the following sections are associated with modulation of the above-mentioned signalling pathway of NF-κB including macrophage polarisation.

The study by Chang et al. [36] demonstrated that phloretin can suppress the level of p65 protein in LPS-stimulated RAW 264.7 cells, an effect coupled with an increased level of phosphorylated IκB-α. This activity not shared by phlorizin was an indicator of the effect of phloretin on the canonical pathway of macrophage activation. The RAW 264.7 cells experimental model employed by Chauhan et al. [43] used *Escherichia coli* (and *E. coli* K1 strain) infection instead of LPS. They have demonstrated that phloretin (50, 100, and 150 μM) can suppress the TLR4-induced NF-κB signalling pathway leading to inhibition of NO and cytokines (TNF-α and IL-6) production. Other studies have further shown that phloretin does not possess a potent antibacterial effect but can prevent biofilm production in bacteria such as in *E. coli* O157:H7 along with inhibition of bacterial attachment to human colon epithelial cells [23]. The lack of antibacterial effect for phloretin, however, needs to be considered with due respect to the strain of bacteria and the concentration used in the experiment as several other studies have reported antibacterial properties for the compound (e.g., [44,45]) or in combination with antibiotics [46]. Wang et al. [47] have reported that phloretin (2–16 µg/mL) treatment attenuated the inflammatory response in the macrophage cell line (J774) co-cultured with *S. aureus* but a direct effect on the activity of the virulence factor, sortase B, was also observed. Sortase B is one of the membrane-bound enzymes that gram-positive bacteria employ to anchor surface proteins to peptidoglycans. It has long been known that sortase B is associated with *S. aureus* virulence and bacterial infection-associated induction of inflammation [48]. The virulence of *Listeria monocytogenes* both in vitro and in vivo has also been shown to be suppressed by phloretin via targeting another virulence factor, sortase A [24].

The effect of phloretin on LPS-induced stimulation of macrophages could be a result of a direct effect on TLRs. Kim et al. [49] assessed TLR2/1 heterodimerization and signalling in RAW 264.7 cells by using a selective agonist, Pam_3_CSK_4_. They have shown that phloretin displays molecular interactions with TLR2/1 and modulates the TLR2 signalling pathway leading to suppressed NF-κB phosphorylation and TNF-α production. Cheon et al. [50] used the *Propionibacterium acnes*-induced skin infection model to examine the effect of phloretin on the TLR-2-mediated inflammatory signalling in human keratinocytes. By measuring secreted embryonic alkaline phosphatase (SEAP) activity induced by either Pam_3_CSK_4_ or by *P. acnes* in HEK-Blue^TM^-hTLR2 cells, which are designed for studying the stimulation of TLR-2 via activation of NF-κB, they were able to confirm the signalling pathway associated with the inhibitory effect of phloretin (5–50 µM). In a similar manor, the *P. acnes*-stimulated levels of TNF-α, IL-1β, and IL-12 as well as phosphorylated c-Jun *N*-terminal kinase (JNK) in HaCaT cells was inhibited by phloretin through inhibition of the TLR-2 signalling pathway. While the study by Chang et al. [36] (see above) demonstrated phlorizin displays weak or no effect when applied directly on LPS-stimulated RAW 264.7 macrophage, positive results were reported for phlorizin metabolites. When tested at low concentrations (1–5 μg/mL), phloretin 4-*O*-β-D-glucuronide, 6-methoxyl-phloretin-2-*O*-β-D-glucuronide, and phloretin-2-*O*-β-D-glucuronide inhibited NO production and iNOS protein expression [51]. Their effect on cytokine expression could however vary and hence a more comprehensive in vivo analysis is needed to assess the potential of phlorizin metabolites as anti-inflammatory agents.

Supporting the role of mitogen-activated protein kinases (MAPK) pathway in the anti-inflammatory effect of phloretin, inhibition of the LPS-stimulated phosphorylation of extracellular signal-regulated kinase 1/2 (ERK1/2), p38, and JNK was reported in the RAW 264.7 cells [36]. These three main kinases (ERK, p38, and JNK) are activated by various inflammatory stimuli and convert the extracellular response to signalling pathways often through crosstalk with the NF-κB-mediated gene activation. The inhibitory effect of polyphenols such as gallic acid and proanthocyanidins against proinflammatory cytokines release and NF-κB activation in the LPS-stimulated RAW 264.7 macrophages has been shown to be mediated through inhibition of the ERK, p38, and JNK [52,53]. When mouse dendritic cells were stimulated by LPS, the TLR4-mediated ROS, MAPKs (ERK, JNK, and p38 MAPK), and NF-κB as well as the production of proinflammatory cytokines and chemokines were shown to be suppressed by phloretin [54]. As shown further in the later sections, phloretin appears to modulate its anti-inflammatory effect partly through altering the proinflammatory mediators-induced upstream cell signalling cascades involving the MAPKs.

To study the anti-inflammatory effect of phloretin in vivo, Huang et al. [55] employed the LPS-induced lung injury model in mice where phloretin (20 mg/kg, intraperitoneal (i.p.)) was shown to suppress the upregulated neutrophil infiltration in lung tissue and reduce the levels of IL-6 and TNF-α in serum and bronchoalveolar lavage fluid. In the lung tissues, phloretin also suppressed the level of activity of MPO and superoxide dismutase (SOD) activity and decreased the gene expression levels of chemokines and proinflammatory cytokines (IL-1β, IL-6, TNF-α, MCP-1, and CCL5), intercellular adhesion molecule-1 (ICAM-1), iNOS, and COX-2 in inflamed lung tissues. The serum level of IL-6 and TNF-α were also suppressed in LPS-treated animals. As with the in vitro data, phloretin also reduced the phosphorylation of NF-κB and MAPK, while promoting the expression of haeme oxygenase (HO)-1 and nuclear factor erythroid 2-related factor 2 (Nrf2) [55]. In the lung model of inflammation induced by *Mycobacteria tuberculosis,* phloretin (2.5 or 5 mg/kg, i.p.) effectively suppressed the levels of TNF-α, IL-1β, and IL-6 in lung tissue [45].

A considerable level of research has been done in the past few years to show that the antioxidant and anti-inflammatory effects of several compounds are mediated through the expression of the Nrf2, which derives macrophage to anti-inflammatory phenotype (M2). The Nrf2 is another master transcription factor that regulates the cellular response to inflammation and oxidative stress. The Nrf2 binds to the regulatory regions (antioxidant response element (ARE)) of target genes to upregulate the expression of cytoprotective or antioxidant enzymes such as phase II detoxification enzymes and stress proteins. By far the most characterised genes/proteins under the regulatory control of Nrf2 are haeme oxygenase-1 (HMOX1, HO-1), glutamate-cysteine ligase, glutathione peroxidase 1 (GPX1), thioredoxin reductase 1 (Txnrd1), NAD(P)H-quinone oxidoreductase 1 (NQO1), glutathione-*S*-transferase (GST), superoxide dismutase (SOD), catalase (CAT), peroxiredoxin (PRDX1), and ferritin. The Nrf2 activity depends on the negative regulator, Kelch-like ECH-associated protein 1 (Keap1, also called electrophile response element), which binds to it and presents it for ubiquitination and subsequent degradation by proteosomes. External stimuli such as ROS-based electrophilic reaction with Keap1 lead to the inactivation of Keap1 and leave the Nrf2 stable or available for nuclear translocation, and transcriptional induction of Nrf2-target genes. Alternative mechanisms of Nrf2 regulation are also known but not discussed herein. For details of Nrf2 regulation in inflammation/oxidative stress, readers should refer to review articles on this topic [56,57,58,59]. In this line, phloretin (50 μM) treatment of mouse bone marrow-derived macrophages have been shown to reduce the inflammatory phenotype of macrophages through the upregulation of the Nrf2-signalling pathway [33]. In macrophages stimulated with phorbol 12-myristate 13-acetate, the levels of ROS and mRNA of proinflammatory genes NOS2, IL-6, COX2, and IL-12 were all shown to be reduced by phloretin, while Nrf2-response genes, HO-1 and NQO1 were activated. Interestingly, Keap1 degradation in macrophages was enhanced by phloretin. Furthermore, phloretin treatment led to the AMP-activated protein kinase (AMPK) phosphorylation and activation in macrophages which was shown to be associated with cell autophagy (see details in Section 12). In an in vivo model, a macrophage-driven experimental autoimmune encephalomyelitis (EAE) was reported to be suppressed by phloretin (50 mg/kg, i.p.). This was evidenced by increased expression of anti-inflammatory and neurotrophic factors, i.e., IL-4, ciliary neurotrophic factor, and insulin-like growth factor-1 in the spinal cord of EAE animals, elevated Nrf2 signalling in the CNS and increased mRNA expression of Nrf2 and its downstream targets, NQO1 and GPX1.

Another experimental model of sepsis used to demonstrate the anti-inflammatory effect of phloretin was induced by caecal ligation and puncture in rats. In this case, phloretin administered at the dose of 50 mmol/kg (i.p.) could ameliorate the increased level of TNF-α in the blood and NF-ĸB p65 in the liver [60]. In vivo, the administration of phloretin also suppressed the phenotypic maturation of the LPS-challenged splenic dendritic cells and decreased the IFN-γ production from the activated CD4 T cells. In RAW 264.7 macrophages exposed to chronic obstructive pulmonary disease (COPD) associated pathogens (*Moraxella catarrhalis* and *Streptococcus pneumoniae*) and nontypeable *Haemophilus influenzae* (NTHi)-induced human bronchial epithelial (HBE) cells, the upregulated TNF secretion was suppressed by phloretin (100 µM) [44]. Other anti-inflammatory effects of phloretin in immune cells include suppression of NO production in concanavalin A-stimulated T lymphocytes and LPS and IFN-γ activated macrophages at doses of 40, 60, and 80 µM [61]. Overall, it appears that the in vitro data observed for phloretin is supported by in vivo evidence using various experimental models. As a summary (see also Section 12), the key features of phloretin in the suppression of inflammation via inhibition of macrophages and other immune cell activation are shown in Table 1.

## 3. Obesity-Associated Inflammation

Phloretin (at concentrations less than 100 µM) is known to inhibit adipogenesis and promote apoptosis in adipocytes in vitro [62,63,64]. In oleic acid-treated HepG2 cells, it also prevented excessive lipid accumulation and decreased the sterol regulatory element-binding protein 1c (SREBP-1c), inhibiting the expression of fatty acid synthase (FAS), while it increased the sirtuin 1 (SIRT1), and phosphorylation of AMPK to suppress acetyl-CoA carboxylase expression thereby suppressing the synthesis of fatty acids [65]. In high-fat-diet (HFD)-fed obese mice, phloretin reduced body weight and fat weight, liver weight and liver lipid accumulation, and improved hepatocyte steatosis. In liver tissue of obese mice, phloretin further suppressed transcription factors of lipogenesis and fatty acid synthase, and increased lipolysis and fatty acid β-oxidation. In addition, phloretin regulated serum leptin, adiponectin, triglyceride, low-density lipoprotein, and free fatty acid levels in obese mice [65]. The role of phloretin in lipid metabolism is, however, complex as it also promotes adipocyte differentiation in vitro and improves glucose homeostasis in vivo by increasing the expression of adipose-related genes or adipogenic markers (peroxisome proliferator-activated receptor-γ (PPARγ), CAAT enhancer binding protein-α (C/EBPα), fatty acid synthase, fatty acid-binding protein 4, and adiponectin such as fatty acids translocase and fatty acid synthase [64]. In this connection, phloretin is known to enhance adipocyte differentiation and adiponectin expression by mechanisms including PPARγ activation as well as modulation of gene expression with the implication of reducing insulin resistance [66]. When RAW 264.7 macrophages were co-cultured with 3T3-L1 cells during adipocytes differentiation, phloretin (and to a very less extent phlorizin) inhibited the adipogenesis-related transcription factors. The phosphorylation of AMPK and the activity of adipose triglyceride lipase and hormone-sensitive lipase were augmented. As an anti-inflammatory compound, phloretin also suppressed the NF-κB and MAPK pathways [67]. Its effects on obesity-associated inflammation are further scrutinised below.

The nonalcoholic fatty liver disease (NAFLD) experimental model is routinely used for the evaluation of therapeutic agents targeting the various components of metabolic dysfunction associated with lipids. By using Huh7 cells exposed to high doses of free fatty acids, Chhimwal et al. [68] have shown that phloretin encouraged autophagy-mediated hepatic lipid clearance and restored mitochondrial membrane potential and redox homeostasis. In the in vivo model of NAFLD using mice subjected to a western diet, a reduction in hepatic histological injury, hepatic lipogenesis, and enhanced fatty acid oxidation were observed in phloretin (50, 100, and 200 mg/kg, p.o.)-treated animals. While improving body weight, oral glucose tolerance, and antioxidant status in the liver (increased levels of SOD, CAT, and decreased lipid peroxidation products, malondialdehyde (MDA) status, a reduction in inflammation was evident as demonstrated by diminished levels of TNF-α and IL-6 in the liver. Beyond the anti-inflammatory effect, phloretin restored the AMPK activity (induction of phosphorylation of AMPK) in the liver of NAFLD and fatty acids-loaded hepatocyte cell lines. The activity of PPARα and its target genes carnitine palmitoyltransferase 1 (CPT1) and CPT2, as well as fatty acids metabolism in the liver, was also augmented by phloretin supplementation in the diet.

In the LPS- and cobalt chloride (CoCl_2_)-stimulated 3T3-L1 adipocytes, phloretin (100 μM), but not phlorizin, suppressed the mRNA expression and secreted levels of IL-6, MCP-1, MCP-3, MIP-1α, and leptin, while increasing adiponectin levels. Phloretin (100 μM) but not phlorizin also reduced ROS accumulation and NF-κB activation in LPS and CoCl_2_-stimulated adipocytes. Another interesting finding was the ability of phloretin-treated adipocytes (conditioned media) to reduce macrophage mRNA expression of the M1 polarization markers, iNOS, IL-6, and TNF-α, and increased expression of the M2 polarization markers (including IL-10, and TGFβ [69]. Linking the antiobesity property or HFD-induced liver injury by phloretin with activation of the Nrf2-mediated cytoprotective pathway was demonstrated in the study by Sampath et al. [70] where an increase in Nrf2 and HO-1 expression were associated with reversal of the HFD-induced pathology in experimental animals.

## 4. Endothelial Inflammation and Injury

Mao et al. [71] assessed the potential of phloretin in ameliorating endothelial injury both in vitro and in vivo using the streptozotocin (STZ)-induced diabetes mouse model. In human umbilical vein endothelial cells (HUVECs) stimulated with high glucose, phloretin (10, 20, or 40 µM) suppressed endothelial damage as evidenced by the reduced level of damage markers such as monocyte chemotactic protein-1 (MCP-1) as well as pro-calcification factors bone morphogenetic protein-2 and receptor activator of NF-κB ligand expression. It also reversed the increased vimentin and decreased CD31 expression both in vitro and in vivo. This effect occurring without alteration in blood glucose levels suggests a mechanism of action unrelated to the modulation of glucose metabolism in diabetic animals. Phloretin (25 or 75 mg/kg, p.o.) further ameliorated endothelial injury and vascular fibrosis in diabetic mice [71]. It also enhanced the activation of the AMPK and upregulated the peroxidase proliferator-activated receptor-γ coactivator-lα (PGC1α) level, while inhibiting the activation of the TGFβ-Smad2-Snail-signalling pathway, which is dependent on AMPK activation as confirmed by inhibitor study [71]. In HUVECs stimulated with TNF-α or IL-1β, the upregulated expression of both at the mRNA and protein levels of vascular cell adhesion molecule-1 (VCAM-1), ICAM-1, and E-selectin was suppressed by phloretin [72]. Consequently, the cytokine-induced adhesiveness of HUVECs and human aortic endothelial cells to monocytes (THP-1 cells) was suppressed by phloretin. The common transcription factor of cytokine genes, NF-κB, was not altered by phloretin but the TNF-α-induced upregulation of another transcription factor in endothelial cells, adhesion interferon regulatory factor 1, was inhibited. Phloretin also inhibited platelet aggregation induced by adenosine diphosphate and thrombin receptor-activating peptide by suppressing the expression of the GPIIb/IIIa complex [72]. By making adducts with methylglyoxal (MGO), phloretin (10 µM) showed an effect against advanced glycation end products (AGEs)-induced cell damage and inflammation in HUVECs [73]. In addition to inhibiting the formation of AGEs by directly trapping MGO, some of the adduct(s) formed were also able to suppress the AGE-induced inflammation (MCP-1 and IL-6 gene expression) and endothelial cell death. The mechanism of action was further shown to be linked to the phosphorylation of MAPKs, including AGEs-receptor for advanced glycation end-products (RAGE) interaction activating the ERK ½ and p38 MAPK, and subsequent activation of NF-κB which were augmented following AGEs-RAGE interaction. These inflammation-associated MAPK signalling cascades appear to be blocked by phloretin in endothelial cells in the same way as that explained for macrophages in Section 2.

In HUVECs exposed to uric acid, the upregulated proinflammatory factors (ICAM-1, MCP-1, and IL-1β) expression both at protein and mRNA levels and endothelial injury were suppressed by phloretin (5–15 µM) [74]. The mechanism is related to its inhibitory effect on the activation of ERK and NF-κB pathways. While reducing GLUT9 expression, it also enhances the uptake of uric acid in endothelial cells. Since GLUT9 knockdown by siRNA inhibited IL-1β and ICAM-1 expression as well as NF-κB/ERK activation, the effect of phloretin may be explained by its effect on GLUT9 inhibition. In this connection, GLUT9 (SLC2A9) is considered a high-capacity uric acid transporter in endothelial cells [75].

One of the antioxidant mechanisms of phloretin explored in endothelial cells is increasing the activity of MnSOD. In phloretin (50 μM)-treated HUVECs, the increased activity of MnSOD was associated with a higher expression level of SIRT3 through phosphorylation of the AMPK [76]. Mainly located in the mitochondria, the SIRT3 has been known to promote endothelial protection during inflammation such as that induced by sepsis [77]. The known effect of phloretin in activating the Nrf2 pathway may also be involved in the protection of endothelial cells from inflammatory damage. For example, upregulated level of expression/activation of Nrf2 and HO-1 by promoting the phosphorylation of AMPK in endothelial cells was shown to be the basis for the protective effect of phloretin from palmitic acid-induced damage [78].

## 5. Lung Inflammation

In the human lung epithelial cells (A549 cell line), the effect of phloretin as an anti-inflammatory agent is similar to those described for macrophages (Section 2). Huang et al. [79] have shown that phloretin (3–100 μM) treatment of A549 cells could alleviate inflammatory conditions induced by IL-1β as evidenced by a reduction in the expression of COX-2, and production of PGE_2_, IL-8, MCP-1, and IL-6. In addition to relieving these potent inflammatory markers of lung inflammation, ICAM-1 gene, and protein expression as well as monocyte adhesion to inflammatory A549 cells were suppressed. This data suggests that phloretin acts as an anti-inflammatory agent by targeting both leucocytes and direct effect on lung epithelial cells. As a mechanism of action, the study [79] also revealed that protein kinase B (Akt) and MAPK phosphorylation as well as NF-κB subunit p65 protein translocation into the nucleus were suppressed by phloretin. Once again, this effect was shown to be limited to phloretin but not phlorizin. As indicated in the preceding section (Section 2), Songyang et al. [35] have shown the potential of phloretin in suppressing acute lung injury via inhibition of the GLUT1-induced glycolysis in macrophages. Phloretin was also reported to suppress GLUT1-dependent glycolysis which is associated with age-dependent fibrogenesis of the lung [80]. In an asthmatic mice model of airway inflammation induced by ovalbumin, phloretin administration (5, 10, or 20 mg/kg, i.p.) reduced goblet cell hyperplasia, eosinophil infiltration (also neutrophil, lymphocyte, and macrophages), and the severity of airway hyperresponsiveness [81]. In addition to suppressing the oxidative stress level (reduction in MDA and increase in glutathione (GSH) levels) in the lung and reduced production of cytokines (IL-4, TNF-α, IL-6, IL-5, and IL-13), chemokines (CCL11 and CCL24) and adhesion molecules (ICAM-1) expression in the lung. The mRNA expression of many inflammatory mediators (CCL11, CCL24, ICAM-1, IL-4 IL-5, IL-13, IFN-γ, MUC5AC, Gob5 (goblet-cell derived protein chloride channel regulator, calcium-activated-1), COX-2, and iNOS) were also suppressed. In the same study [81] in vitro using TNF-α and IL-4-activated human lung epithelial cell line (BEAS-2B cells), phloretin (10 and 30 µM) was shown to suppress the production of chemokines (CCL11, CCL24, and CCL26) as well as epithelial cells adhesion to monocytes (THM-1 cells). Given the compound also inhibited ROS production in BEAS-2B cells, a dual effect on both lung oxidative stress and inflammation was evident for the compound. As a mechanism of action, an increased level of expression of Nrf2 in lung cells was also induced by phloretin. These data overall support that already discussed (Section 2) for phloretin-mediated protection of the lung from LPS-induced injury in vivo [55].

The anti-inflammatory potential of phloretin was assessed both in vitro and in vivo using an experimental model of airway inflammation induced by cigarette smoke [82]. When the human lung mucoepidermoid cells (NCI-H292 cell line) were exposed to cigarette smoke extract (CSE), the expression of mucin 5ac (MUC5AC) and IL-1β were greatly enhanced, and phloretin suppressed this induction in a dose-dependent manner (1–5 µM). The CSE-induced phosphorylation of epidermal growth factor receptor (EGFR), ERK, and P38 was also inhibited by phloretin. In mice treated with phloretin (10 or 20 mg/kg, i.p.), the effect of CSE in lung histological changes, mucus hypersecretion, MUC5AC expression level, inflammatory cell infiltration in the lungs, and phosphorylation of EGFR, ERK, and P38 were suppressed. Respiratory pathogens (*Haemophilus influenzae*) induced chronic obstructive pulmonary disease (COPD) in mice could also be suppressed by phloretin (0.157% in a diet for a week) as evidenced by reduced bacterial burden, inflammatory score in the lungs, and expression of a neutrophil chemoattractant, chemokine (C-X-C motif) ligand 1 (CXCL1) [44]. In this study [82], the pathogen-induced mucin production was also suppressed by phloretin. In RAW 264.7 macrophages exposed to COPD associated pathogens (*Moraxella catarrhalis* and *Streptococcus pneumoniae*) and nontypeable *Haemophilus influenzae* (NTHi)-induced human bronchial epithelial (HBE) cells, the enhanced TNF secretion was suppressed by phloretin (100 µM) [44]. The direct effect of phloretin on bacteria should not also rule out as a mechanism contributing to the inhibition of pathogenic bacteria-induced lung inflammation. For example, human alveolar epithelial cell (A549 cells) damage caused by *S. aureus* in vitro could be ameliorated by phloretin (2–16 µg/mL) perhaps through a mechanism including a direct effect on bacteria such as an effect on the activity of the virulence factors, as shown for sortase B [82].

In view of the potential effect of phloretin in tuberculosis-associated lung inflammation, Jeon et al. [45] employed the LPS-stimulated human dendritic cells or interferon-γ-stimulated lung fibroblast MRC-5 cells assay model in vitro. In this system too, phloretin (10 or 20 µM) could reduce cytokine production (e.g., IL-1β, IL-12, and TNF)-α) as well as mRNA expression of inflammatory mediators (e.g., IL-1β, IL-6, IL-12, TNF-α, and matrix metalloproteinase (MMP)-1). The mechanism linking this activity in terms of signalling pathway was also investigated, with phloretin demonstrated to suppress the phosphorylation of p38 MAPK and ERK. Furthermore, in LPS-stimulated lung inflammation, the level of pro-inflammatory cytokines (TNF-α, IL-1β, and IL-6) in lung tissues was suppressed by phloretin (2.5 or 5 mg/kg, i.p.).

## 6. Arthritis

The collagen-induced rheumatic arthritis model in mice was used to establish the anti-inflammatory activity of phloretin (50 or 100 mg/kg, p.o.) in vivo [83]. The obvious therapeutic marker was the reduction in disease severity associated with joint inflammation and cartilage- and bone destruction. The reduction in the level of proinflammatory cytokines (TNF-α, IL-6, IL-1β, and IL-17) and decreased oxidative stress (MDA and ROS) further suggest the therapeutic potential of phloretin in this disease model. Furthermore, the human osteoarthritis chondrocytes stimulated with IL-1β was used as an in vitro model of the assay to evaluate the effect of phloretin [84]. It has been shown that phloretin (10, 30, and 100 μM) could suppress the production of NO, PGE_2_, TNF-α, and IL-6, as well as the mRNA expression level of COX-2, iNOS, MMP-3, MMP-13, and disintegrin and metalloproteinase with thrombospondin motifs 5 (ADAMTS-5). The degradation of aggrecan and all the observed effects appear to be linked to the inhibition of the IL-1β-stimulated phosphorylation of phosphoinositide 3-kinases/Akt (PI3K/Akt) and activation of NF-κB. These effects of phloretin could also be reproduced in the mouse model of osteoarthritis as it prevented the destruction of cartilage and the thickening of subchondral bone, amelioration of synovitis, and decreased the expression of MMP-13, while increasing the expression of collagen-II. Overall, inflammatory markers including cytokines, COX, iNOS, and MMPs appear to be regulated by phloretin in arthritis through modulation of signalling such as NF-κB and the PI3K/Akt. In the latter case, the PI3K signalling has been shown to promote pro-inflammatory cytokine production such as IL-6 through NF-κB activation downstream of Akt [85]. The effect of natural antioxidants such as curcumin in inhibiting the inflammatory response (e.g., activation of NF-κB activation and induction of iNOS and inflammatory cytokines release) has been shown to be associated with down-regulation of the PI3K/Akt signalling [86].

## 7. Gut Inflammation

By using the standard ulcerative colitis experimental model in mice induced by dextran sulfate sodium (DSS), Wu et al. [87] demonstrated the anti-inflammatory effect of phloretin (60 mg/kg p.o. daily for 7 days) in vivo. The novel feature of this study was faecal microbiota transplantation (FMT) or transferring faecal microbes (10^9^ CFU/mL) from phloretin-treated diseased mice by gavage to non-phloretin-treated diseased mice. This FMT, as well as phloretin treatment, ameliorated ulcerative colitis by improving pathological markers (e.g., reversing the shortening of colon length and increased Muc2 mRNA and Claudin-1, zonula occludens-1 (ZO-1) protein expressions) and colon inflammation (suppression of cytokines including TNF-α, IL-6 and IL-1β, NF-κB and NOD-like receptor family pyrin domain containing 3 (NLRP3) inflammasome activation) and oxidative stress (increase the levels of SOD and GSH and decreasing MDA) markers in colonic tissues. This study which also demonstrated the reversal of the change in the gut microflora population induced by ulcerative colitis supports the hypothesis that some of the phloretin’s effects as an anti-inflammatory agent may be associated with modulation of the gut microbiota structure/population. Further evidence substantiating the link between gut microbiota dysbiosis and ulcerative colitis and the reversal of the disease’s pathology by phloretin came from the study by Liu et al. [88]. In DSS-induced ulcerative colitis in mice, treatment with phloretin (100 mg/kg, p.o. for three weeks) was shown to reverse the disease score, inflammatory markers (NF-κB activation), and reverse the change in microbiota population of the gut. In an experiment using a lower dose of phloretin (60 mg/kg, p.o.), the DSS-induced ulcerative colitis score in mice was shown to be correlated with normalising the composition of microbes and their metabolites [89].

In the acetic acid-induced ulcerative colitis experimental model in rats, administration of phloretin (50 mg/kg, p.o.) for three days was able to restore the biochemical abnormalities in the plasma (plasma alkaline phosphatase (ALP) and lactate dehydrogenase (LDH) level) and inflammatory (downregulate MPO, NO, and eosinophil peroxidase level as well as ICAM-1 gene) and oxidative (increase in GSH level) biochemical markers in colon tissues [37]. Lee at al [23] studied the potential of phloretin in colon inflammation in rats induced by pathogenic bacteria *E. coli* O157:H7. In addition to the reduction in *E. coli* O157:H7 biofilm formation, the compound (10 or 50 µM) suppressed bacterial attachment to human colon epithelial cells (HT-29 cells) and the TNF-induced inflammatory response in vitro such as monocyte (U937) attachment to colonic cells. This anti-inflammatory effect in vitro could be reproduced in animal models (20 mg/kg, p.o. for 5 days) when colitis was induced in rats using 2,4,6-trinitrobenzene sulphonic acid (TNBS) as revealed by body weight, colon weight, and MPO activity. In the *Salmonella typhimurium* infection model in mice, phloretin (25, 50, or 100 mg/kg, p.o.) could reverse body weight loss and attenuated weight loss and colon length shortening as well as inflammatory cell infiltration [90]. The treatment also suppressed the systemic spread of the infection along with the expression of proinflammatory cytokines (TNF-α, IL-β, and IL-6) in colon tissues as well as NF-κB p65 and TLR4 expression. Similar to its protective effect in the gut induced by other stimulants, phloretin also ameliorates oxidative stress (increased activity of SOD and GSH level and reduced MDA level) in colonic tissues along with improved expression of ZO-1 and occludin [90].

In the study by Wang et al. [91] using the LPS-induced damage to bovine rumen epithelial cells (BRECs) in vitro, phloretin (100 µM) was demonstrated to suppress the expression of pro-inflammatory cytokines (IL-1β, IL-6, IL-8, and TNF-α) and chemokines (CCL2, CCL5, and CCL20). This response of LPS was mediated via the TLR4 signalling pathways that involve NF-κB p65 and ERK1/2 (p42/44) which were inhibited by phloretin. The LPS-induced decrease in the expression of claudin-related genes (ZO-1 and occludin) was also reversed by phloretin suggesting normalisation of both cellular structure and inflammatory damage by LPS. Another in vitro model employed for assessing the potential effect of phloretin in the gut was that using the IL-1β-stimulated myofibroblasts of the colon CCD-18Co cell line [92]. In this case, phloretin (10–50 μM), but not phlorizin, decreased the synthesis of PGE_2_ and IL-8. In the bovine serum albumen (BSA)/glucose and BSA/MGO assays, both phloretin and phlorizin inhibited the formation of the AGEs in a dose-dependent manner (0.01 to 1.0 mM). Overall, phloretin appears to ameliorate gut inflammation induced by a variety of agents. In addition to reversing the damage induced in the gut tissues, inflammatory markers are suppressed, and the normal microbiota structure and population restored.

## 8. Hepatic and Renal Dysfunction

The hepatoprotective effect of phloretin has been demonstrated under various in vivo experimental models including overdose intake of acetaminophen [28], choline [93], D-galactosamine (D-GalN) [30], and arsenate [94]. These effects which are also shown in renal protection by toxic agents (e.g., by Singh et al. [94]) mostly demonstrated the antioxidant mechanism of therapeutic potential of phloretin. The focus herein is to highlight the inflammatory mechanisms of hepato- and renoprotective effects.

Beyond the hypoglycaemic effect of phloretin, beneficial effects in various diabetes-related pathologies have been documented. In the STZ and HFD-induced diabetes model in Apolipoprotein E knockout (ApoE^−/−^) mice, phloretin as a dietary supplement (20 mg/kg) has been shown to reduce polyuria, proteinuria, and glomerular histopathological changes [14]. This protective effect against renal dysfunction without significant alteration in the blood glucose levels includes improvement in glomerular basement membrane thickening, podocyte foot process effacement, and restoration of levels of nephrin and podocin. In the adenine/potassium oxonate-induced hyperuricemia in mice, phloretin administration (50 or 100 mg/kg, p.o.) reduced the level of serum blood urea nitrogen (BUN), urinary albumin to creatinine ratio, tubular necrosis, extracellular matrix deposition, and interstitial fibroblasts [95]. Notably, phloretin reduced renal infiltration of inflammatory cells, cytokines such as NLRP3 and IL-1β release, mitochondrial ROS, and morphological lesions. Another interesting finding of this study was that phloretin caused a significant reduction in both mRNA and protein levels of GLUT9 under inflammatory conditions (but not in normal kidneys). On the other hand, in vitro, phloretin (10 or 15 µM) suppressed the expression of NLPR3, IL-1β, and IL-18 under LPS or uric acid stimulation in HK-2 (human kidney 2, proximal tubular) cells. As described for endothelial cells, GLUT9 has been shown to play a key role in renal reabsorption of uric acid in vivo [96].

In the study using cisplatin-induced nephrotoxicity in mice, phloretin or phlorizin (50 and 100 mg/kg, i.p.) normalised the biochemical markers of the disease such as antioxidant (SOD activities and GSH level) and oxidative stress (MDA levels) status coupled with normalisation of pathological features such as structural damage of tubular and glomeruli in the kidney [97]. In terms of inflammation, the cisplatin-induced TNF-α, IL1β, and NF-κβ mRNA expressions were shown to be suppressed by phloretin in kidney tissues. It is worth noting that, although both compounds were active, the reduction in the induced cytokine expression was higher for phloretin than phlorizin. Another marker of cellular damage suppressed by the compounds was the expression of heat shock protein 70 expressions, suggesting the trend of change in kidney tissues towards normalisation [97].

There are also studies showing the hepatoprotective effects of phlorizin. For example, in the hepatoprotective study using methotrexate in rats, phlorizin (40 or 80 mg/kg, p.o.) was shown to suppress hepatic injury (histopathological evidence) coupled with the reduction in alanine transaminase (ALT), aspartate aminotransferase (AST), and lactate dehydrogenase (LDH), TNF-α and COX-2 levels, and increase in hepatic total antioxidant capacity (TAC), GSH, GST, CAT, and SOD levels [98]. Hence, it can be summarised that both phloretin and phlorizin can suppress oxidative stress and inflammation induced by experimental agents in the liver and kidney tissues.

## 9. Cardiac Inflammation

The cardiotoxic effect of arsenic trioxide was used as a model to study the protective effect of phloretin in an in vitro assay using the cardiomyocyte cell line, H9c2 cells [99]. The cardiac toxicity markers including Ca^2+^ overload, expression of calcineurin, nuclear factor of activated T cells (NFATC), and apoptosis markers (BAX and JNK) were inhibited, while cytoprotective factors (BCL-2, IGF1, Akt, RAF1, ERK1, and ERK2) were increased. As an anti-inflammatory mechanism, the expression levels of proinflammatory cytokines (IL-2, IL-6, MCP-1, IFN-γ, and TNF-α) and NF-κB were inhibited by phloretin. By using the STZ-induced diabetic mouse model, Ying et al. [100] studied the cardioprotective effect of phloretin (20 mg/kg, p.o. daily for 8 weeks). The hyperglycaemia-induced upregulation of biochemical markers suggesting myocardial injuries such as LDH, creatine kinase MB (CK-MB) and AST) were reduced. Reduction in the hypertrophy-related genes in the heart of diabetic animals treated by phloretin was also coupled with inhibition of the higher level of expression of atrial natriuretic peptide (ANP), brain natriuretic peptide (BNP), and β myosin heavy chain (β-MyHC). While the level of ROS in cardiac tissues was suppressed by phloretin, the mRNA expression levels of HO-1, NQO-1, and glutamate-cysteine ligase were upregulated. It also suppressed the expressions of collagen-1, connective tissue growth factor (CTGF), and TGFβ. In an in vitro study using high glucose-stimulated H9c2 cells, the authors also showed phloretin (10 μM) effectively suppressing oxidative stress (decreasing MDA and increasing SOD activity) by increasing the genes expression of Nrf2, NQO-1, and HO-1. The hyperglycaemia-induced increases in hypertrophy factors such as ANP, BNP, and β-myosin heavy chain and fibrosis factors (TGF-β and CTGF) were also inhibited as assessed by their genes expression. Making the Nrf2 available by obstructing the interaction between Nrf2 and Keap1 via direct binding to Keap1 has been suggested as the possible mechanism of action, a hypothesis supported by molecular docking studies. The Arsenic trioxide-induced toxicity in cardiac H9c2 cells could also be ameliorated by phloretin (2.5 and 5 µM) through an antioxidant mechanism linked to the increased transcriptional activity of Nrf2 [101]. In agreement with the above-mentioned study [100], the same pharmacological effect on the cardioprotective effect of phloretin was reported by Ying et al. [102] using the STZ-induced diabetes model in mice and high glucose-stimulated H9c2 cells. In this case, the compound was also shown to decrease hyperglycaemia and improved diabetes-induced increases in AST, CK-MB, and heart weight. The other data were similar to that reported above [100] except for the upregulated expression of silent information regulator 2 homolog 1 (SIRT1) in cardiac cells treated by phloretin under high glucose in vitro. Their data have further shown that the knockdown of SIRT1 aggravates the high glucose-induced inflammation, hypertrophy, and fibrosis mRNA level in H9C2 cells. All these data suggest that phloretin can protect cardiac cells from oxidative stress and inflammation through mechanisms including modulation of the Nrf2, NF-κB, and SIRT1 pathways.

## 10. Neuroinflammation

The 1-methyl-4-phenyl-1,2,3,6-tetrahydro pyridine (MPTP)-induced Parkinson’s disease (PD) model in mice was employed to explore the neuroprotective effect of phloretin [103]. Beyond improving the behavioural symptoms of PD and striatal dopamine level, the increased expression of TNF-α, IL-1β, and IL-6 in brain tissues were suppressed by phloretin treatment (5 mg/kg, p.o.). Moreover, microglial and astrocytes markers expression (glial fibrillary acidic protein, allograft inflammatory factor 1 (Iba-1), iNOS, and COX2), which were increased by MPTP, were also suppressed by phloretin. Given the small dose used in this study, phloretin can be considered a potent inhibitor of neuroinflammation under PD conditions. In the D-GalN-induced aging model of mice, the neuroprotective effect of phlorizin (20 and 40 mg/kg, p.o.) was shown by improvement in memory functions and reversal of histopathological alterations and biochemical parameters of oxidative stress (MDA content and SOD and CAT activities in the serum, liver, and brain) and inflammatory markers (NF-κB activation and IL-1β expression in brain tissues) [104]. Correlation between these changes and microbiota alterations as well as restoration also suggests the microbiota-brain axis of neuroprotection. It would be interesting to know whether phloretin could also show similar effects in this experimental model. In the LPS-induced cognitive impairment in mice, phlorizin (10–20 mg/kg, oral) also restored memory functions along with reversing the decreased level of antioxidants (SOD and GSH), the brain-derived neurotropic factor (BDNF) and cholinergic transmission (increased acetylcholinesterase (AChE)) in the hippocampus and cortex [105]. On the other hand, phlorizin reversed the increased levels of inflammatory/oxidative markers (TNF-α, IL-6, and MDA). In a sporadic rat model of Alzheimer’s disease induced by injection of Aβ_25–35_, phloretin has been shown to improve the spatial memory formation and retention and antioxidant markers as well as the level of TNF-α in the brain homogenates [106].

In a diabetic neuropathy study induced by STZ, phloretin (50 or 25 mg/kg, i.p.) or in combination with duloxetine (15 mg/kg duloxetine and 25 mg/kg phloretin, i.p.) was shown to ameliorate the thermally induced hyperalgesia, sciatic nerve oxidative stress markers (tissue MDA, NO, SOD activity and GSH levels); and sciatic nerve TNF-α and IL-6 levels [107]. This, together with the normalization of histopathological and structural changes, suggests the potential of phloretin as a neuroprotective agent. In HFD- and STZ-induced diabetes models in mice, phlorizin (10–20 mg/kg, p.o.) was shown to alleviate depression symptoms, reversed the decline in GSH, BDNF, and its receptor (TrkB), cyclic AMP-responsive element-binding protein (CREB) and ERK level [108]. Inflammatory markers were not, however, assessed in this study.

The link between the activation of the Nrf2 and the neuroprotective effect of phloretin was demonstrated in the study by Liu et al. [109]. They have shown that oxidative stress injury after cerebral ischemia/reperfusion in rats induced by middle cerebral artery occlusion was ameliorated by phloretin via upregulating Nrf2 mRNA and protein expression thereby reversing the decreased level of SOD, GSH, and GSH peroxidase (GPx), and the increased MDA levels in brain tissues. The neuroprotective effects of phloretin in vitro are also known. For example, phloretin has been demonstrated to offer a protective effect in SH-SY5Y cells from rotenone-induced toxicity [109]. Overall, both phloretin and its glycoside, phlorizin, appear to ameliorate neuronal injury, oxidative stress, and neuroinflammation through multiple mechanisms including the previously explained modulation of the NF-κB and Nrf2 pathways.

## 11. Skin Inflammation

As indicated in Section 2, phloretin relieved the *P. acnes*-induced skin inflammation in various experimental models [50]. Kum et al. [110] studied the anti-inflammatory effects in HaCaT cells based on *P. acnes*-induced inflammatory mediators release where phloretin was demonstrated to suppress the release of PGE_2_ and inhibit COX-2 expression. This and human clinical studies in a 4-week trial suggest that the compound has the capacity to suppress *P. acnes*-induced skin inflammation. The study by Wu et al. [111] further employed a dinitrochlorobenzene (DNCB)-induced dermatitis experimental model in mice where phloretin (50 and 100 mg/kg, p.o. for 21 days) was shown to alleviate the disease markers. This includes downregulating the induced epidermal thickening process and infiltration of mast cells into the lesion regions, and reduction in the levels of histamine and pro-inflammatory cytokines (IL-6, IL-4, thymic stromal lymphopoietin, IFN-γ and IL-17A) in the serum. As a mechanism of action, the MAPK and NF-κB pathways were suppressed by phloretin in dermal tissues. The over-proliferative ability of splenocytes in vitro from the DNCB-treated animals was also suppressed in the phloretin-treated group along with suppression of the elevated frequencies of Th1 (CD4^+^IFN-γ^+^), Th2 (CD4^+^IL-4^+^), and Th17 (CD4^+^IL-17A^+^) cells population.

## 12. General Discussion

In the various sections of this review (Section 2, Section 3, Section 4, Section 5, Section 6, Section 7, Section 8, Section 9, Section 10 and Section 11), the anti-inflammatory potential of phloretin that has been established through studies both in vitro and in vivo are outlined. Indeed, phloretin does also possess various other pharmacological effects including anticancer properties. Readers should bear in mind that carcinogenesis and the process of cancer metastasis including angiogenesis share some aspects of inflammation biology. Hence, the reported anti-inflammatory mechanism of phloretin could also be relevant to its anticancer properties. The focus of this section is however to summarise the anti-inflammatory mechanism of phloretin that accounts for its diverse other effects in vitro and in vivo.

One line of evidence in the anti-inflammatory effect of phloretin lies in its ability to inhibit GLUT1, which is a major glucose transporter in macrophages stimulated by LPS [112]. The GLUT1 overexpression in macrophages has also been shown to increase glucose uptake, glycolysis, and the expression of pro-inflammatory cytokines (e.g., TNF-α, IL-1β, and IL-6). The alteration of macrophages to a pro-inflammatory phenotype (M1) that is characterized by excessive proinflammatory cytokine and ROS production is thus mediated by GLUT1 activation [32]. Accordingly, a variety of agents that downregulate GLUT1 expression suppress macrophages activation (e.g., [113,114,115]). LPS treatment also resulted in a 7- and 4-fold increase in the GLUT1 protein content in Kupffer and endothelial cells respectively [116]. Thus, amelioration of inflammatory response by suppressing the blockade of glycolysis in macrophages is also evident in other cell types [117]. As discussed in Section 2, the association between GLUT1 and LPS-induced inflammation both in vitro and in vivo has been established and phloretin mediates some of its anti-inflammatory effects via inhibition of GLUT1 [35]. As uric acid transporter in endothelial and other cells, GLUT9 also plays a role in inflammation which is inhibited by phloretin [75].

**Figure 2:**
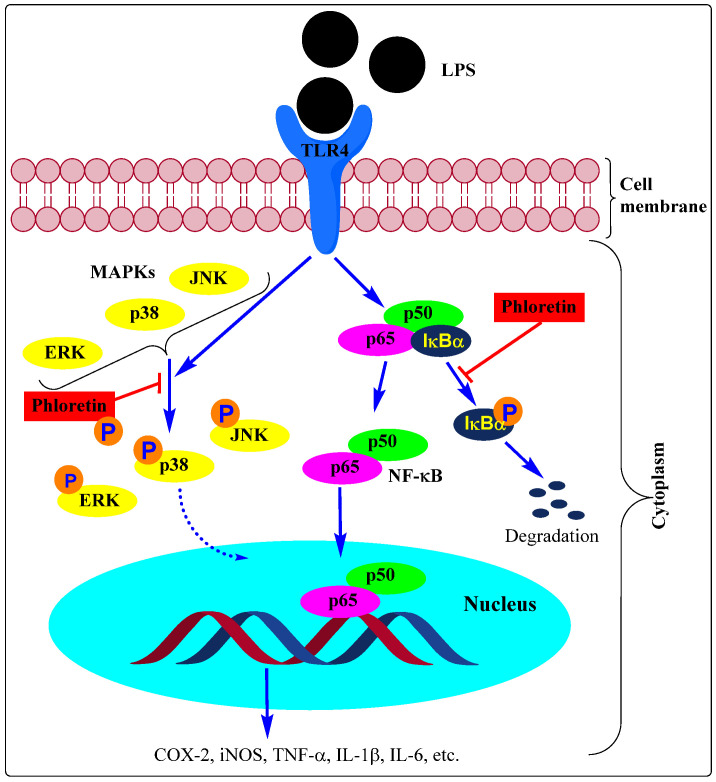
Anti-inflammatory effect of phloretin through inhibition of phosphorylation of MAPKs and IκBα. Most of the studies on the activation of inflammatory mediators from immune cells employed LPS which interacts with its receptor, TLR4. While a direct effect on TLR1/2 has been suggested, most of the data indicate an inhibitory effect on NF-κB mobilization to the nucleus. The LPS response shown by blue arrows includes the degradation of the NF-κB inhibitory protein, IκBα, via promoting its phosphorylation. This frees the p65/p50 to mobilise to the nucleus and activate inflammatory target genes such as COX, iNOS, and cytokines including TNF-α, IL-1β, and IL-6. Phloretin has been shown the suppress the NF-κB activity by inhibiting IκBα degradation (see red lines). The exact mechanism of how the MAPK activity leads to the activation of proinflammatory genes has not yet been fully established but the key steps after induction by inflammatory agents such as LPS is the phosphorylation of p38, ERK, and JNK which have been shown to be targeted by phloretin (see red lines). Note that other pro-inflammatory agents such as TNF-α, IL-1β, and IL-6 can also activate the MAPK and NF-κB pathways by interacting with their cell-surface receptors.

We have a very long list of compounds that suppress inflammation through inhibition of the NF-κB activation pathway in immune cells thereby inhibiting the induction of proinflammatory genes such as COX, iNOS, cytokines, chemokines, and adhesion molecules. The effect of phloretin as an anti-inflammatory agent via alleviating inflammatory signals in a variety of cell types discussed in this review was mediated through modulation of the NF-κB activation pathway (e.g., [36,43,55,60,67,73,79,84,87,91,103]. Studies using various immunologically relevant human cell lines (human cell lines (DLD-1 (colorectal), T84 (colon), MonoMac6 (monocytic), Jurkat (T-cells)) also revealed that phloretin can inhibit the expression of proinflammatory genes by repressing NF-κB and proinflammatory cytokines [118]. The expression of these genes is also linked to the activation of the MAPK pathway and the effect of phloretin as an anti-inflammatory agent has been shown to be linked to the inhibition of the phosphorylation of the three major MAPKs, p38 MAPK, ERK, and JNK (e.g., [36,44,54,55,73,79,111,119]). These pathways of signalling and modulation by phloretin are shown in Figure 2.

The other major pathway of the anti-inflmmmatory mechanism for phloretin is the activation of the Nrf2 transcription pathway. This common mechanism of anti-inflammatory and antioxidant response in various cells appears to be shared by many cytoprotective agents. In many experiments, the Nrf2/ARE/HO-1 axis is extensively explored as with modulation by phloretin discussed in this communication under the various sections [33,70,78,94,100,101,120]. The Nrf2 signalling has also been shown to be relevant in the anti-inflammatory effect of phlorein in non-immunological cell types. For example, in LPS-stimulated retinal pigment epithelial cells (ARPE-19 cell line), phloretin was shown to suppress the release of proinflammatory cytokines (IL-6, IL-8, and VEGF) through a mechanism associated with an increased level of Nrf2 expression [120].

Evidence is now emerging in support of the PI3K/Akt pathway of NF-κB activation which is targeted by phloretin. Readers should bear in mind that this pathway is far too complex and in some cases, inhibition of the pathway displays protective effects while in others, it worsens the inflammation (see review, [121]). More research is thus needed to ascertain the significance of either enhancing or suppressing the PI3K/Akt pathway by phloretin.

**Figure 3:**
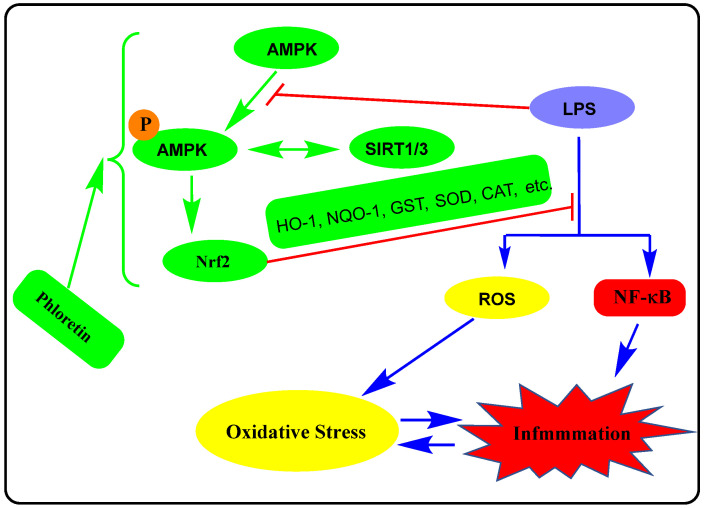
The crosstalk between AMPK, Nrf2, and SIRT1 pathways in the anti-inflammatory effect of phloretin. The activation of AMPK and SIRT1 are closely related in the regulation of metabolism and pathologies such as oxidative stress and inflammation. Activation of AMPK by phloretin via its phosphorylation as well as increased expression of SERT1 have been associated with its anti-inflammatory effect and modulation of lipid metabolism such as inhibition of lipid accumulation. This activity was further shown to be related to the induction of Nrf2 that suppresses both the oxidative stress and inflammatory pathways induced by a variety of agents including LPS. Increased SERT3 activity by phloretin is also reported. The red line shows the inhibitory response.

Another interesting line of evidence for the anti-inflammatory effect of phloretin was linked to the modulation of AMPK signalling. The AMPK severs as a metabolic sensor in cells by responding to changes to ADP:ATP and AMP:ATP ratios. Increased level of ATP consumption and/or decreased ATP synthesis activates AMPK leading to the phosphorylation of key proteins that stimulate ATP-generating pathways (fatty acid oxidation, mitochondrial biogenesis, and muscle glucose transport) while inhibiting the ATP-consuming anabolic pathways (protein translation, fatty acid, and cholesterol synthesis) [122]. In addition to playing a key role in carbohydrate and lipid metabolism in health and disease, the AMPK signalling pathways play a key role in the regulation of inflammation. Generally, a decrease in AMPK activity in cells is associated with increased inflammation, while agents such as phloretin that activate AMPK can inhibit inflammatory responses induced by different stimuli. Hence, AMPK agonists can downregulate the immune response and ameliorate inflammation in vivo [123] as with many compounds that activate AMPK primarily via its phosphorylation [124,125]. Moreover, activation of AMPK can stimulate the Nrf2 signalling pathway that has been suggested as an anti-inflammatory mechanism for a variety of agents [126,127,128,129]. The cross-talk between the activation of AMPK through its phosphorylation and SIRT1 has been known in lipid metabolism regulation and pathologies mediated by oxidative stress and inflammation. Both SIRT1 and AMPK have been shown to be involved in the anti-inflammatory effect of phloretin in the HFD-inflammation model in cultured cells [65]. This is further shown to be correlated with the Nrf2 pathway of anti-inflammatory/antioxidant mechanisms in high-glucose mediated damage in cardiac cells [100,102]. The link between activation of the AMPK pathway and NF-κB suppression by phloretin has been established in macrophages [67], endothelial injury [76,78] antioxidants, and anti-inflammatory effect in vivo [68], under endothelial injury and vascular fibrosis in diabetic mice [71]. It has been shown that SIRT1 and NF-κB activation have antagonistic activity in the regulation of metabolism (e.g., gluconeogenesis, glycolysis, fatty acid oxidation, and synthesis, oxidative phosphorylation, mitochondrial biogenesis, etc.) including insulin resistance and inflammation [130,131,132]. Moreover, the effect of AMPK in metabolism and inflammation has been shown to involve the activation of SIRT1 [133] and Nrf2 signalling (see review by Petsouki et al. [134]). The effect of drugs in the AMPK/SIRT1/NF-κB axis observed in many drugs is thus evident in phloretin too which promotes AMPK/SIRT1 activation while downgrading NF-κB. The overall activity of phloretin in this signalling is depicted in Figure 3.

While the direct antibacterial effect of phloretin is weak to moderate, the compound has been shown to suppress biofilm formation and pathogenicity by targeting virulence factors. Its effect on bacterial toxins such as endotoxin/LPS by a mechanism including inhibition of their interaction with cell receptors such as TLRs appears to be another mechanism of action for its anti-inflammatory effects associated with a bacterial infection. Perhaps the most prominent effect of phloretin related to bacteria is its effect on the gut microbiota. Many inflammatory diseases in the gut such as ulcerative colitis or neurodegenerative diseases are associated with the change in the gut microbiota. The increase in the population of pathogenic bacteria (e.g., *Proteobacteria* such as *E. coli*), hand in hand with decreases in beneficial bacterial species (e.g., *Lactobacillus*) in the gut, is termed dysbiosis. Modulation in their composition (e.g., in the ratio of *Firmicutes/Bacteroidetes bacteria)* is associated with inflammatory disease progression. Hence, restoration of the gut microbiota by approaches such as dietary intervention, probiotics/prebiotics, FMT, supplementation with gut microbiota metabolites, and drug therapy have now become common research topics in therapeutics of anti-inflammatory diseases. Review articles discussing the role of the gut microbiota in pathologies such as heart [135,136], brain [137,138], rheumatoid [139,140], liver [141,142], inflammatory bowel [143,144], cardiovascular [145,146], metabolic disorder [147,148], respiratory [149,150] including COPD [151], kidney [152,153], diseases among others, are available. In this connection, the reported positive modulatory effect of phloretin as anti-inflammatory agents, coupled with the improvement of the gut microbiota dysbiosis, is interesting and includes experimental models of in silico studies [154], DSS-induced ulcerative colitis [87,88], modulate GLP-1 secretion [155], and FMT model [87].

Increasing the uptake of phloretin in biological systems to improve its efficacy received some attention in recent years. This has also been tried out in inflammation research [156] but most of the studies are related to absorption from the skin given the wide application of the compound in skin products. Thus, polymeric nanocapsules loaded with phloretin [157], hydrophilic, and other lipophilic formulations [158], and absorption from skin lotions [159] have been studied. By using mixed polymeric-modified self-nanoemulsions, Wang et al. [160] have shown that the water solubility of phloretin can be enhanced 3000-fold, its bioavailability by 7.9-fold, and it is in vivo anti-inflammatory effect by 6.8-fold. The application of fast-dissolving nanofibers [161] and nanoparticles [162] including chitosan nanoparticles [163] for drug-resistant cells in cancer therapy using phloretin formulation has also been explored. Moreover, various other approaches are now adopted to formulate phloretin for a better therapeutic efficacy outcome and more research in this field will solve its limitation in bioavailability.

Further lead improvement of phloretin and large-scale isolation requires a review of plant sources from which these chalcones have been isolated. So far, the various parts of the apple tree (*Malus domestica*) have been used as sources for high-yield isolation of phloretin and/or its glycoside, phlorizin. Literature outlining the extraction of these compounds from apple tree leaves, bark, and fruits including pulp, peal, and apple pomace are rich. As already discussed, a simple hydrolysis of phlorizin can yield phloretin—i.e., any plant source rich in phlorizin can also be used as a source of phloretin. Several *Malus species* including *Malus doumeri* [164] and *Malus pumila* [165] have also been studied both for their phytochemistry related to phloretin/phlorizin and biological activities. Furthermore, phloretin and phlorizin are detected in many families of plants (see review by Gosch et al. [166]) including strawberries [167], *Cynomorium songaricum* [168], grapefruits (*Vitis vinifera*) [169], pomegranate (*Punica granatum*) [170], and cranberries (*Vaccinium macrocarpon)* [171] but with low concentrations. The various derivatives identified in these plants would offer further lead optimization through structural-activity relationship studies. Hence, there is an abundant natural source of phloretin for further lead development if it is to be taken as a drug candidate through an anti-inflammatory mechanism of action.

## 13. Conclusions

Phloretin, originally identified as a natural product from the apple tree and as a metabolic product of phlorizin, has diverse pharmacological effects. While the use of its glycoside (phlorizin), mostly known in the diabetes field, can release phloretin in vivo, there seems to be a distinct difference in the pharmacological effects of the two compounds. In this communication, the superior effect of phloretin as an anti-inflammatory agent when assessed under various cellular and animal models is highlighted. Phloretin appears to alter the various signalling pathways of inflammation including NF-κB, MAPK, Nrf2, and AMPK among others. Through diverse effects it ameliorates the expression of proinflammatory cytokines, adhesion molecules, chemokines, nitric oxide, and prostaglandins. It also ameliorates the disease-associated changes in the microbiota population or dysbiosis. The major limitation of phloretin remains poor bioavailability which appears to be improved by using novel formulations. Through such research and perhaps lead optimisation via chemical modification as productively demonstrated for phlorizin in diabetes therapy, the therapeutic potential of phloretin for inflammation can be released in the future.

## Figures and Tables

**Table 1:** Summary of the effects of phloretin and related compounds on macrophages and other immune cells activation.

Cellular Model	Treatment (Dose)	Main Outcome	Reference
Mouse bone marrow-derived macrophages	Phloretin (50 μM)	Activates Nrf2 and suppresses the inflammatory phenotype of macrophages; reduces NO production and mRNA levels of pro-inflammatory genes (NOS2, IL-6, COX2, and IL-12); effect not seen in activated Nrf2-deficient macrophages; stimulates autophagy in an AMPK-dependent manner; activates the Nrf2 pathway through autophagy-mediated Keap1 degradation.	Dierckx et al. [33]
LPS-stimulated primary mouse peritoneal macrophage	Phloretin (25 and 50 μM)	Decreases the mRNA level of IL-1β and TNF-α; inhibits the protein and mRNA upregulation of GLUT1 (but not GLUT3 and GLUT4) induced by LPS; inhibits glycolysis in LPS-treated macrophages in a GLUT1-dependent manner.	Songyang et al. [35]
LPS-stimulated murine RAW 264.7 macrophage	Phloretin and phlorizin (3–100 μM)	At an effective dose of 10 μM, phloretin inhibits the levels of NO, PGE_2_, IL-6, TNF-α, iNOS, and COX-2; suppresses the nuclear translocation of NF-κB subunit p65 proteins; decreases phosphorylation in MAPK pathway; no effect observed for phlorizin up to 100 μM.	Chang et al. [36]
LPS-stimulated RAW 264.7 cells	Phloretin (EC_50_ values of 5.2 μM)	Inhibits MPO and iNOS.	Arya and Kanthlal [37]; Van Thu et al. [38]
RAW 264.7 cells infection by virulent *E. coli* K1 strain	Phloretin(5–150 μM)	Reduces NO, ROS, TNF-α, and IL-6 production; downregulates the expression of *E. coli*-induced COX-2, NF-κB pathway, and HO-1 in a concentration-dependent manner.	Chauhan et al. [43]
COPD pathogen-induced RAW 264.7 macrophages	Phloretin (0.1–1 mM)	Reduces TNF secretion.	Birru et al., 2021 [44]
TNF-α-stimulated RAW 264.7 cells	Phloretin (1–20 μM)	Inhibits TLR2/1 heterodimerization; reduces TNF-α; and IL-8; exhibits micromolar binding affinity to TLR2.	Kim et al. [49]
LPS-stimulated RAW 264.7 cells	Phloretin derivatives (1–5 μg/mL)	Phloretin 4-*O*-β-D-glucuronide, 6-methoxyl-phloretin-2-*O*-β-D-glucuronide, and phloretin-2-*O*-β-D-glucuronide inhibi NO production and iNOS protein expression.	Zhao et al. [51]
LPS-stimulated mouse dendritic cells	Phloretin (50 μM)	Suppresses the production of ROS and proinflammatory cytokines; inhibits MAPKs (ERK, JNK, and p38 MAPK), and NF-κB.	Lin et al. [54]
Mouse T lymphocytes and macrophages	Phloretin (40, 60, and 80 μM)	Inhibits the proliferation of T lymphocytes; inhibits the expression of CD69 and CD25; induces cell cycle arrest at G0/G1 phase; reduces NO production of LPS-stimulated macrophages; reduces phagocytosis rate of macrophages.	Lu et al. [61]

Abbreviations: COPD, chronic obstructive pulmonary disease; COX, cyclooxygenase; ERK, extracellular signal-regulated kinase; HO-1, haem oxygenase 1; iNOS, inducible nitric oxide synthase; IL, interleukin; JNK, c-Jun *N*-terminal kinase; LPS, lipopolysaccharide; MAPK, mitogen-activated protein kinases; MPO, myeloperoxidase; NF-κB, nuclear transcription factor κ-B; NO, nitric oxide; Nrf2, nuclear factor erythroid 2-related factor 2; PGE_2_ prostaglandin E_2_; ROS, reactive oxygen species; TLR, Toll-like receptor; TNF, tumour necrosis factor.

## Data Availability

Not applicable.
